# Protein profile of extracellular vesicles derived from adult *Parascaris* spp.

**DOI:** 10.1186/s13071-024-06502-3

**Published:** 2024-10-10

**Authors:** Vishnu Manikantan, Nichol E. Ripley, Martin K. Nielsen, Sriveny Dangoudoubiyam

**Affiliations:** 1 Department of Comparative Pathobiology, College of Veterinary Medicine, Purdue University, 625 Harrison Street, West Lafayette, IN 47907 USA; 2 Maxwell H. Gluck Equine Research Center, Department of Veterinary Science, University of Kentucky, Lexington, KY 40503 USA

**Keywords:** Exosomes, *Parascaris*, Proteome, Ascarids, Extracellular vesicle, Nematode, Horse, EV-like vesicles

## Abstract

**Background:**

*Parascaris* spp. represent a significant threat to equine health worldwide, particularly in foals. The long-term survival of parasites in the host necessitates persistent modulation of the host immune response. Intercellular communication achieved through the exchange of molecules via extracellular vesicles (EVs) released from the parasite could be a crucial factor in this regard. This study aimed to isolate and characterize EVs released by adult male and female *Parascaris* worms and conduct a proteomic analysis to identify sex-specific proteins and potential immunomodulatory factors.

**Methods:**

Live adult *Parascaris* worms were collected, and EVs were isolated from spent culture media using differential ultracentrifugation. Nanoparticle tracking analysis and transmission electron microscopy confirmed the size, concentration, and morphology of the isolated EVs. Proteins within the isolated EVs were analyzed using mass spectrometry-based proteomics (LC–MS/MS).

**Results:**

Proteomic analysis revealed a total of 113 proteins in *Parascaris* EVs, with several proteins showing homology to known helminth exosome proteins and exhibiting immunomodulatory functions. Sex-specific differences in EV protein composition were observed, with a distinct abundance of C-type lectins in female EVs, suggesting potential sex-specific roles or regulation. Gene Ontology (GO) and Kyoto Encyclopedia of Genes and Genomes (KEGG) pathway analyses revealed metabolic pathways shared between male and female *Parascaris* EVs, as well as differences in signal transduction, and cell growth and death pathways, indicating sex-specific variations.

**Conclusions:**

These findings imply that *Parascaris* EVs and their protein cargo are complex. This data potentially opens avenues for discovering innovative approaches to managing and understanding helminth infection.

**Graphical Abstract:**

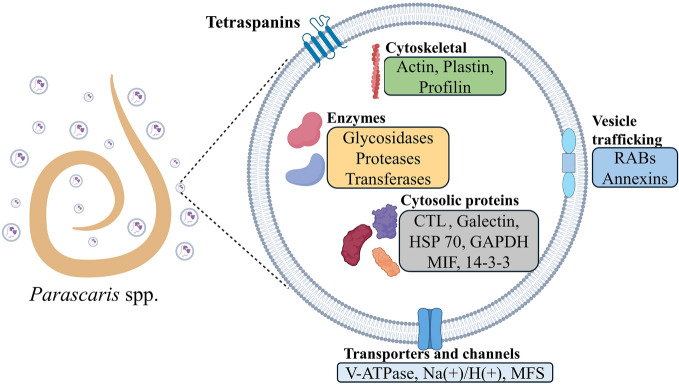

**Supplementary Information:**

The online version contains supplementary material available at 10.1186/s13071-024-06502-3.

## Background

*Parascaris* is an intestinal roundworm that infects horses, especially foals, globally. Two species, the predominant *Parascaris univalens,* and the less frequently encountered *P. equorum*, have been recognized based on their karyotype differences [1, 2]. Being morphologically and biologically indistinguishable, these equine ascarids are referred to as *Parascaris* spp. Infection occurs when horses ingest embryonated *Parascaris* eggs from their surroundings. Once the eggs hatch in the small intestine, the larvae move through the liver and lungs before re-entering the small intestine to mature into adult worms. The migrating *Parascaris* larvae can cause severe damage to the lungs and liver, leading to inflammation, hemorrhage, and edema [3]. Approximately 10–15 weeks after the onset of infection, mature female worms in the small intestine release a large number of eggs in the horse's feces, contaminating the surrounding environment and causing further infections for years to come. Affected young foals exhibit various clinical signs, such as ill thrift, dull coat, stunted growth, colic, diarrhea, and respiratory distress [3]. Small intestine impaction from adult worms is a significant concern and can require hospitalization and surgery or may even result in fatalities [1].

Numerous studies have demonstrated that nematodes release excretory-secretory (ES) products into the host milieu that serve various purposes for the parasite [4] and play key roles in host-parasite interactions. The recent discovery of extracellular vesicles (EVs) in nematode ES products and their analysis revealed marked variability in their size, content, and function, adding another layer of complexity to the host-parasite interaction [5]. Extracellular vesicles are phospholipid bilayer membrane-bound spherical particles classified based on their origin and biogenesis. Most research has focused on the cargo of exosomes (30–100 nm) and microvesicles (100–1000 nm) [6]. Initially, considered to be a carrier of waste products [7], EVs are now recognized for their crucial role in intercellular communication by transporting diverse functional molecules such as proteins, lipids, and nucleic acids (mRNAs, microRNAs, and other noncoding RNAs). Parasite-derived EVs have gained importance as immune modulators but have also been speculated to play a role in parasite-parasite communication [8].

In recent years, parasitic nematodes such as *Ascaris suum* [9]*, Anisakis pegreffii* [10], *Haemonchus contortus* [11], *Teladorsagia circumcincta* [12]*, Heligmosomoides polygyrus* [13]*, Nippostrongylus brasiliensis* [14], *Trichuris muris* [13]*,* and *Brugia malayi* [15] have been shown to release EV-like vesicles when cultured in vitro. These studies also indicate that EV proteins are immunoreactive, participate in host-parasite interactions and immunomodulation, support parasite survival, contribute to pathogenesis, and that EVs can also transfer potential virulence factors [8].

Although existing knowledge underscores the importance of EVs in these processes, sex-specific divergence in EV cargo composition among parasitic helminths has largely remained an unexplored territory. However, the proteomic analysis of *Brugia malayi* EVs revealed that EVs from female worms had a more diverse proteome than EVs from male *Brugia* worms. Notably, functional proteins with immunomodulatory roles were more abundant in the female EVs [15] suggesting that the ability of the parasite to manipulate the host immune response may be parasite sex dependent. These distinct characteristics or sex-specific effects on the host might provide a better understanding of the mechanism by which the parasites can thrive and endure in their host's hostile environment. With regard to *Parascaris* spp., the excretory-secretory proteins of the larval stage were studied [16] with a focus on identifying immunoreactive ES proteins, but *Parascaris* EVs and their contents remain uninvestigated. In the present study, we isolated and analyzed the proteins from extracellular vesicles released into the culture medium by the adult male and female *Parascaris* spp.

## Methods

### Collection of adult *Parascaris* spp and their invitro maintenance

Live adult *Parascaris* *univalens* [2] were collected from the small intestine of foals necropsied at the Gluck Equine Research Center, University of Kentucky, with approval from the university's Institutional Animal Care and Use Committee under protocol no. 2021–3879. These adult male and female worms were washed at least six times with phosphate-buffered saline (PBS) at 37 °C. Three replicates, each comprising five adult worms, either males or females only, were transferred into T75 culture flasks containing 50 ml culture media and incubated at 37 °C with 5% CO_2_. Dulbecco's Modified Eagle's Medium (DMEM) (Cytiva, USA) supplemented with 100 µg/ml streptomycin and 100 U/ml penicillin served as the culture medium to maintain the viability of the live adult worms. The spent culture medium in the flasks at 24 h was collected and processed for extracellular vesicle isolation, separately for each replicate.

### Extracellular vesicle isolation

To isolate EVs from the spent culture media collected at 24 h, a series of differential centrifugations followed by ultracentrifugation steps were performed with slight modifications to the protocol previously described by [17]. Briefly, lower-speed centrifugation steps were sequentially employed to remove eggs (300 × g, 10 min), cell debris (2000 × g, 10 min), and large EVs (10,000 × g, 30 min). Between each step, the supernatant was collected, and the next centrifugation was performed. The supernatant obtained after the removal of larger EVs was stored at −80 °C. To isolate EVs, the supernatant was thawed and centrifuged at 120,000 × g for 90 min in 25 × 89 mm polyallomer tubes using a SW 32 Ti swinging bucket rotor in an Optima MAX ultracentrifuge (Beckman Coulter, California, USA). The EV pellet was washed once with ice-cold PBS and subjected to a final spin at 120,000 × g for 90 min. The supernatant was discarded, and the EV pellet was resuspended in 500 µL of PBS for downstream quantification and proteomics analysis. All centrifugation steps were performed at 4 °C, no brakes were applied, and the samples were kept on ice throughout the EV isolation process. The resuspended EVs were stored at − 80 °C.

### Nanoparticle tracking analysis and transmission *electron* microscopy

Visualization and characterization of the isolated EVs were performed using nanoparticle tracking analysis (NTA) and transmission electron microscopy (TEM) of the negatively stained EVs. For NTA, aliquots of EVs were diluted in particle-free PBS (filtered through a 0.1 μm membrane) to obtain measurable concentrations of suspended EVs (30–100 particles per field). A NanoSight LM10 Nanoparticle Analysis System (Malvern Instruments, Malvern, UK) equipped with a 60 mW laser operating at 405 nm was used to acquire and analyze the EVs in each field for 60 s. Each diluted EV replicate from male and female *Parascaris* spp. was analyzed thrice. The mean size and concentration of the EVs in each sample were determined by calculating the averages from the histograms generated during the NTA [18].

The services of the Life Science Microscopy Facility at Purdue University were used to perform negative staining of the isolated EVs. Briefly, 1.5 μl of the EV suspension was deposited onto a copper mesh EM grid and washed three times with water. Subsequently, the grid was incubated with 1% phosphotungstic acid for 60 s. The excess staining solution was blotted off, and the grid was imaged under an FEI TECHNAI G2 20 transmission electron microscope.

### Quantification and silver staining of the extracellular vesicle proteins

*Parascaris* EVs were lysed by resuspending them in 0.2% sodium dodecyl sulfate (SDS) [19]. The protein concentration was estimated using a Qubit™ assay (Thermo Fisher Scientific, USA). A preliminary analysis of the quality and complexity of the *Parascaris* male- and female-derived EV proteins was performed by silver staining [20] of two micrograms of EV proteins separated by 12% SDS–PAGE [21].

### Mass spectrometry of the *Parascaris* EVs

In preparation for the proteomics analysis, the *Parascaris* adult male and female-derived EVs (individual replicates) were lysed in VK05 Precellys tubes containing 350 μL of 100 mM HEPES–KOH and 0.5 mm glass beads using a Precellys^®^ Evolution homogenizer (Bertin Technologies, France). Three cycles of 20 s at 6000 rpm with a 30-s pause between each cycle were used to disrupt the EVs. The protein concentration of the EV lysate was estimated using the bicinchoninic acid (BCA) assay kit. An appropriate volume of the EV lysate was precipitated with cold acetone (−20 °C). The protein pellet obtained was resuspended in 8 M urea, reduced, alkylated, and digested overnight with trypsin. The resulting peptides were desalted using Pierce^™^ C18 micro spin columns (Thermo Fisher Scientific, MO, USA) as per the manufacturer’s recommendations. The eluate was dried in a Vacufuge Plus centrifuge concentrator (Eppendorf, Hamburg, Germany) and resuspended in 3% acetonitrile and 0.1% formic acid for LC‒MS/MS analysis.

The male and female-derived EV lysate samples were analyzed by reverse-phase HPLC–ESI–MS/MS using a Vanquish Neo UHPLC System (Thermo Fisher Scientific, MO, USA) coupled to an Orbitrap Exploris 480 MS (Thermo Fisher Scientific, MO, USA) equipped with a Nanospray Flex Ion Source (Thermo Fisher Scientific, MO, USA). The purified peptides were separated on a 15 cm analytical column (75 μm id) packed with 2 μm 100 Å PepMap C18 medium (Thermo Fisher Scientific, MO, USA) with a gradient elution spanning 130 min. Mass spectrometry was conducted in data-dependent mode, with full scan MS spectra acquired in the 350–1600 m/z range and MS/MS scans executed using high-energy C-trap dissociation.

### MaxQuant analysis

MaxQuant software version. 2.0.3.0 (http://www.maxquant.org), with its built-in Andromeda search engine, was used to analyze all LC‒MS/MS data. The MS/MS spectra were searched against the *Parascaris univalens* (Taxon ID-6257, Assembly: ASM225920v1, Bioproject ID: PRJNA386823, sequences: 31,353, release 2023–1) sequences in the UniProt database for protein identification and relative abundance profiling. The database search was performed with a minimum length of six amino acids, a precursor mass tolerance of 10 ppm, and an MS/MS fragment ion tolerance of 20 ppm. For the enzyme specificity of trypsin, up to two missed cleavages were allowed. Oxidation of methionine and N-terminal acetylation were defined as variable modifications, and carbamidomethylation of cysteine was defined as fixed modifications for database searches. Peptide quantitation was performed using “unique plus razor peptides.” The false discovery rate of peptide and protein identification was set at 1%. Proteins labeled as contaminants or reverse hits and proteins identified in all three replicates with at least 2 MS/MS counts were included for further analysis. Proteins containing indistinguishable peptides were grouped to satisfy the principles of parsimony. Relative protein abundances were measured based on the normalized spectral abundance factor (NSAF).

### Bioinformatics analysis

Protein descriptions for UniProt accession numbers and Gene Ontology categories were predicted using the software Blast2GO basic version 5.2.5 [22]. Each match was manually validated and confirmed using InterPro [23], which detects the presence of conserved protein domains. The Exocarta database [24] was searched for reported associations with small EVs in mammals based on protein names or Blast2GO descriptions. Signal P analysis was performed to identify classically secreted proteins [25]. Gene Ontology analysis was carried out using WEGO [26]. KEGG Orthology (KO) terms were assigned to the identified proteins using the online tool GhostKOALA [27].

## Results

### Characterization of EVs released by *Parascaris* spp.

**Fig. 1:**
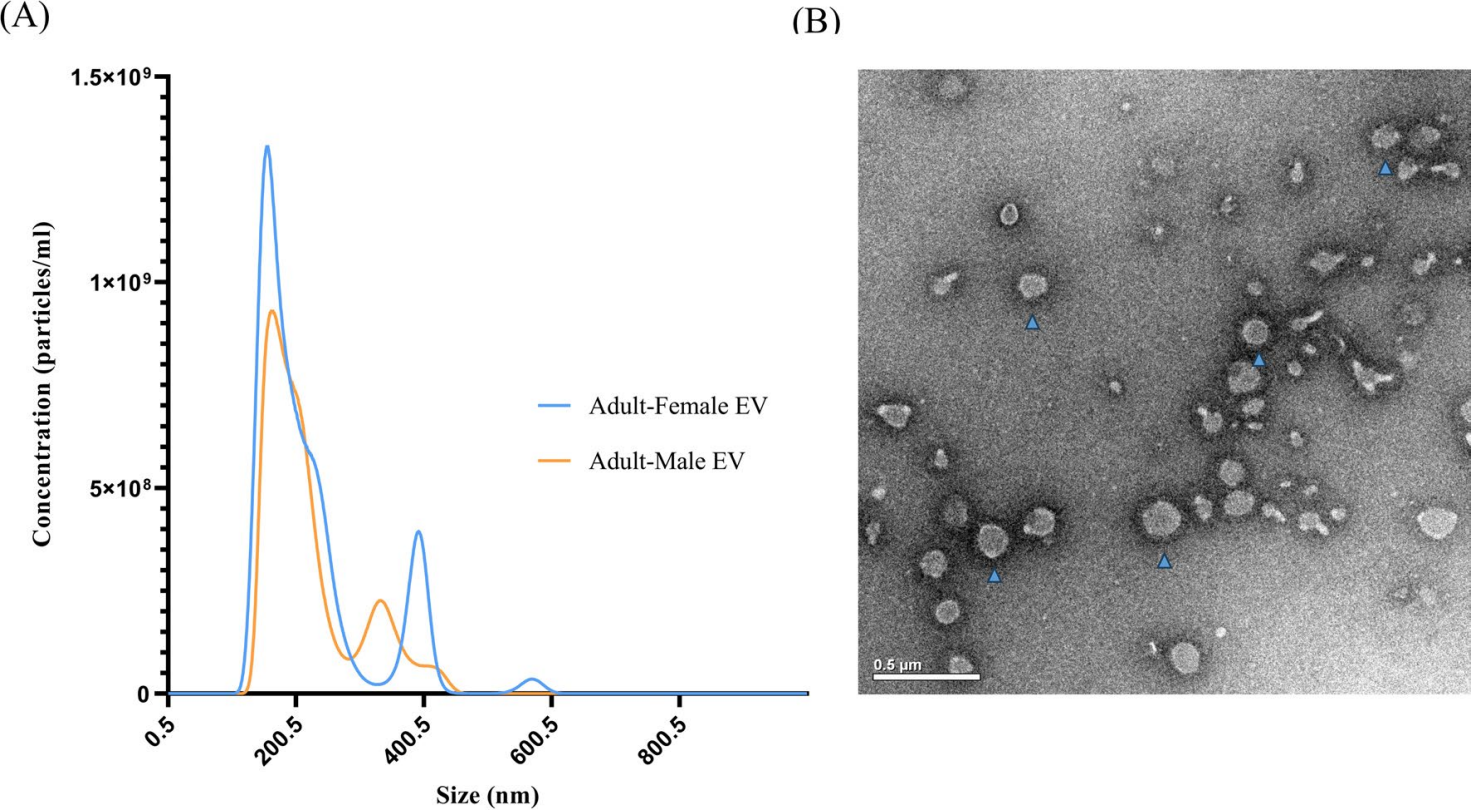
Characteristics of extracellular vesicles released by adult male and female *Parascaris* spp. EVs isolated from the spent culture medium at 24 h of incubation of the worms analyzed by (**A**) NTA show a predominant size of 200 nm, and (**B**) TEM shows their spherical shape (blue arrowhead)

**Fig. 2:**
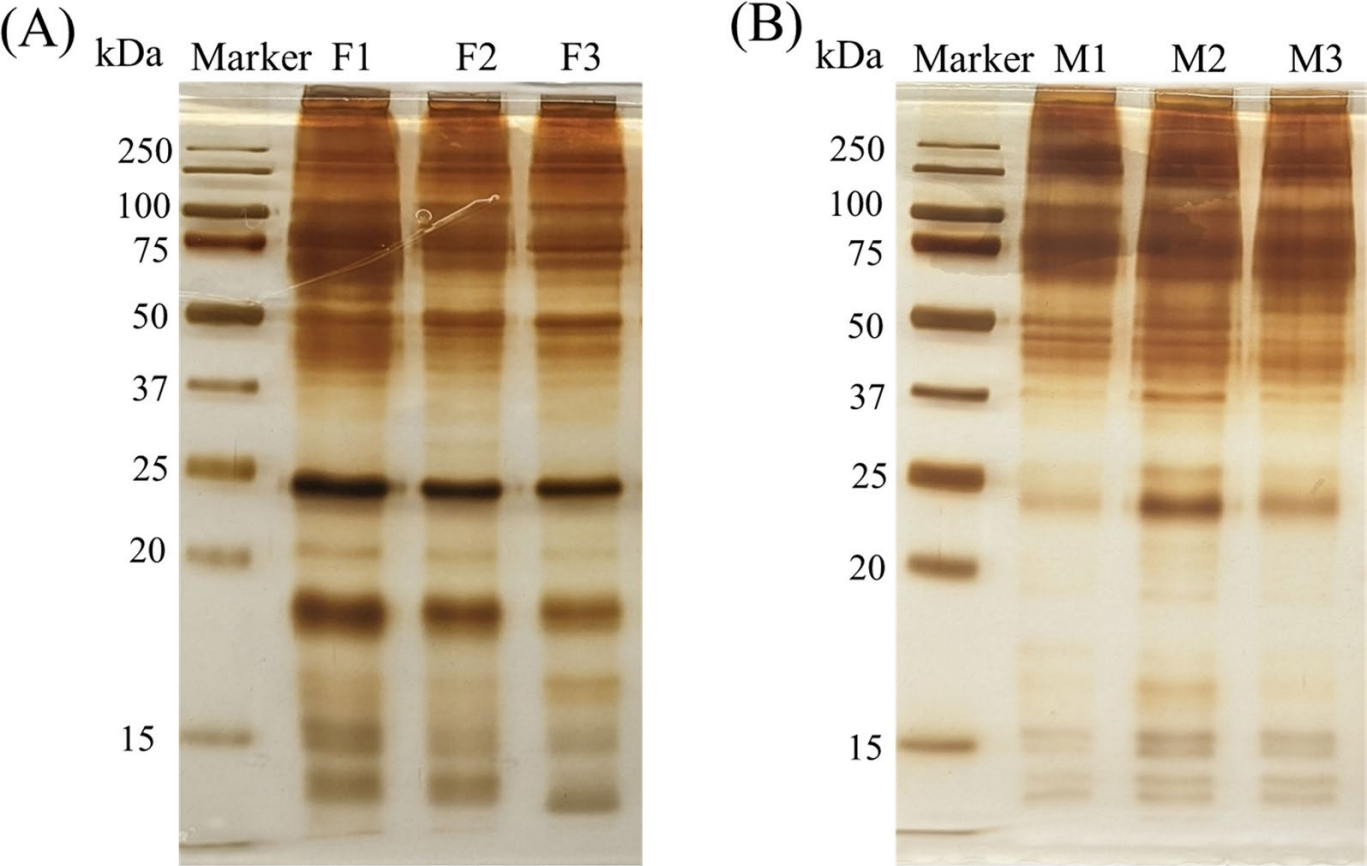
Protein profile of extracellular vesicles released by the male and female *Parascaris* spp. The EV lysate preparations separated on 12% SDS-PAGE and silver stained show heterogeneous composition. Additionally, the EV protein profiles of (**A**) females (F1, F2, F3) and (**B**) males (M1, M2, M3) are distinct, especially around ≤ 25 kDa

The NTA results indicated that most EVs exhibited vesicle sizes ranging from 80 to 200 nm, which aligns with the typical size characteristics of small EVs (Fig. 1A). The average concentration of EVs from adult males was 1.01 × 10^11^ ± 2.83 × 10^10^ particles/ml and had a mean size of 200.4 ± 17.4 nm. The EVs from females had a concentration of 1.35 × 10^11^ ± 1.06 × 10^10^ particles/ml and a mean size of 218.2 ± 7.1 nm. Negative staining and TEM analysis of EVs revealed a consistent rounded shape with diameters ranging from approximately 50 to 200 nm (Fig. 1B), corroborating the measurements obtained through NTA. Silver staining of EV lysates separated by SDS-PAGE showed protein bands in the molecular weight range of 15 to 250 kDa. Additionally, differences in the protein content of EVs from male and female *Parascaris* were evident among proteins migrating at approximately ≤ 25 kDa (Fig. 2). Specifically, the EV lysate from female *Parascaris* spp. contained two predominant bands at ~ 24 and 18 kDa, while EVs from male *Parascaris* exhibited a major band at 24 kDa alone and, based on the staining intensity, appeared to exist at lower levels.

### Proteomic analysis reveals *Parascaris* EV proteins

LC‒MS/MS proteomic analysis performed on the EVs isolated from the spent culture media in which the adult female and male *Parascaris* were maintained identified 113 proteins, and their identities were established through a combination of BLAST and InterPro homology searches (Additional file 1: Table S1). For 105 of the 113 proteins identified, the top hit was an ascarid, with 85 of them showing similarity to a protein of *Toxocara canis*, a dog ascarid. The InterPro descriptions of the identified proteins included proteases, glycosylases, hydrolases, C-type lectins, and galectins, among others. Enzymes accounted for 32% of these identified proteins. Among the 113 identified EV proteins, 20 were among the top 100 EV proteins in the Exocarta database (Additional file 1: Table S1). Signal P analysis revealed that 34 of the 113 proteins had a signal peptide. Several proteins commonly observed in EVs, such as those associated with EV biogenesis or vesicle trafficking (e.g., annexin, tetraspanin family members, and Rab family members), heat shock proteins, and 14-3-3, were also detected in *Parascaris*-derived EVs. Notably, proteins such as HSP70, 14-3-3, annexin, glutathione-S-transferase, and glyceraldehyde-3-phosphate dehydrogenase (GAPDH), which are commonly found in helminth exosomes were identified in these EVs [9, 10]. Most research on nematode EV proteins has focused on their immunomodulatory role. Our analysis of the proteome of *Parascaris* adult EVs revealed proteins with putative roles in immunomodulatory activity or associations with virulence based on their identity to proteins in related parasites (Table 1). These include serine proteases, fatty acid-binding proteins, galectins, annexins, heat shock proteins, etc.

**Table 1:** Putative immunomodulatory proteins identified in the extracellular vesicles derived from adult male and female *Parascaris* spp.

*Parascaris* spp. UniProt ID	Protein name*	Putative function	Related nematodes and Reference
A0A914ZVF6	14–3-3 protein	suppress production of IL-4, NO, and cell proliferation of the PBMCs	*Haemonchus contortus* [59]
A0A914ZR58, A0A914ZTY0	Annexin	Promotes apoptotic cell engulfment	*Caenorhabditis elegans* [60]
A0A915AP20, A0A915ANV1, A0A915AMH, A0A915BT47	Galectin	apoptosis of activated T cells, especially Th1 cells	*Toxascaris leonina* [61]
A0A915AGS2, A0A915C515, A0A914ZJE2	GST	Th2-type polarization and eosinophil recruitment Essential protein during the invasion, development, and survival	*Trichinella spiralis* [62]
A0A915C3P7	HSP 70	Parasite invasion and pathogenesis	*Strongyloides stercoralis* [63]
A0A915BQX1	MIF	decreases the production of TNF-α, IL-1β and IL-12	*Haemonchus contortus* [46]
A0A915AKF4, A0A915AMS5, A0A915ALF1, A0A915B3J3, A0A915AHV5, A0A915AMT1, A0A915AG65, A0A915BM98, A0A915APJ9, A0A915A960, A0A915B0V2	CTL	interfere with the mucin and binding to host ligands essential for development and reproduction	*Toxocara canis* [64] *Haemonchus contortus* [65]
A0A914ZT10	GAPDH	inhibit complement activation and membrane attack complex formation	*Haemonchus contortus* [38]
A0A915BBF6	FAB proteins	interactions with cell surface receptors to retrieve lipids	*Ascaris suum* [66]
A0A915BYM3	Serine Proteases	nutrition, invasion, and immune evasion degradation of mucins within the mucus barrier	*Trichuris muris* [57, 67]

^*^GST Glutathione S Transferase, *HSP* Heat Shock Protein, *MIF* Macrophage Inhibitory factor, *CTL* C-Type Lectin, *GAPDH* Glyceraldehyde 3-phosphate dehydrogenase, *FAB* Fatty Acid Binding

### Sex-specific EV proteins

**Fig. 3:**
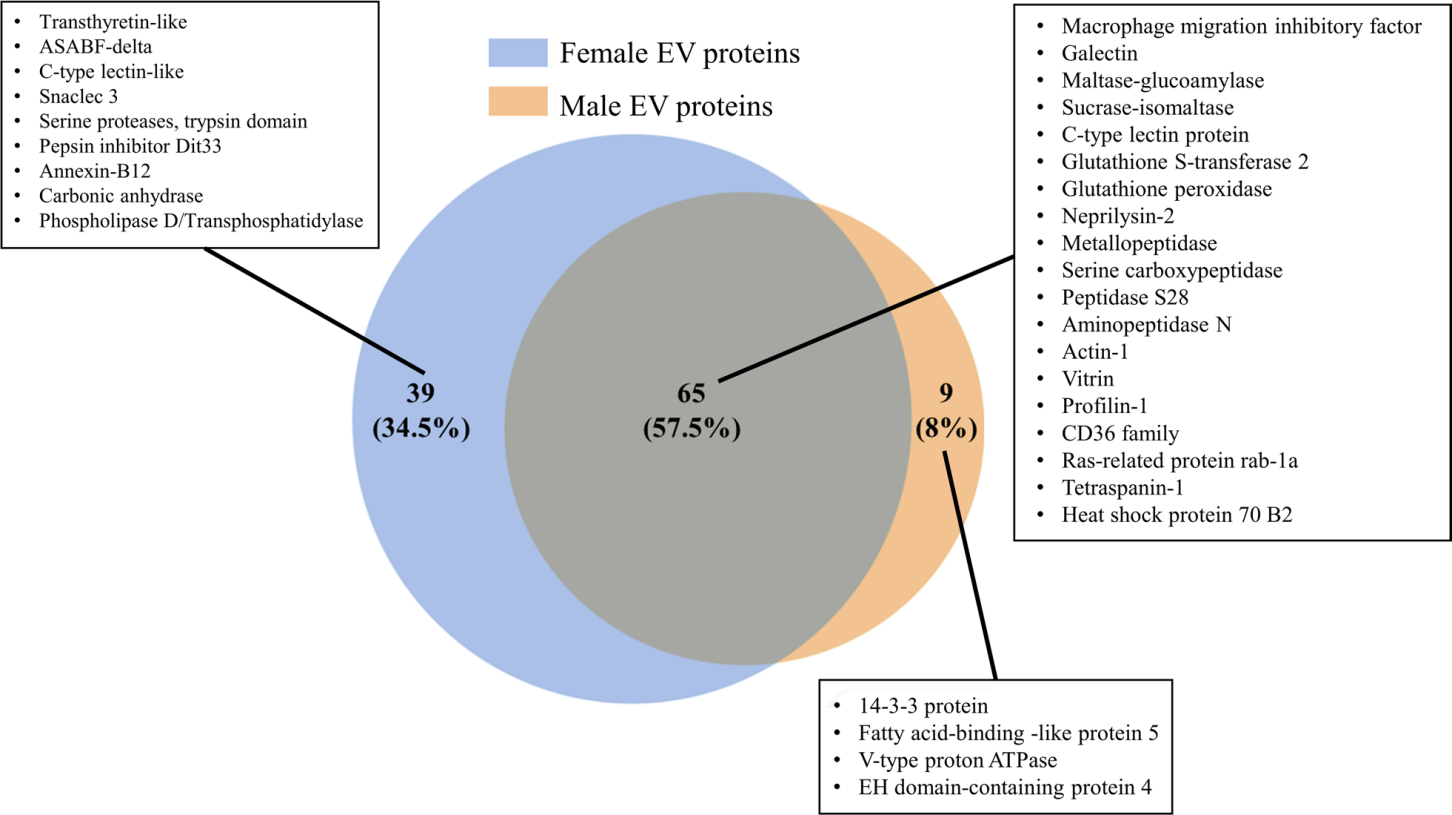
Venn diagram illustrating the proteins associated with extracellular vesicles (EVs) derived from adult male and female *Parascaris* spp. A selection of the identified proteins are provided (the complete list is available in Additional file 2 Table S1 and S2)

Of the 113 extracellular vesicle (EV) proteins identified, 65 were found to be shared between male and female *Parascaris* spp. Meanwhile, 39 proteins were exclusively found in females, with 9 proteins uniquely present in males (Fig. 3, Additional file 2: Table S1). The top ten most abundant proteins of the extracellular vesicles of male and female *Parascaris* spp. calculated on the basis of the NSAF [28] are presented in Table 2. C-type lectins and enzymes of glycoside hydrolase family 31 were the most abundant proteins in both sexes. Notably, some C-type lectins showed a marked predominance in female EVs.

**Table 2:** Top 10 abundant proteins of the extracellular vesicles derived from adult male and female *Parascaris* spp

Uniprot ID	NCBI Blast Description	Accession Number	InterPro Description	InterPro ID	NSAF
Adult female-derived EVs					
A0A915AMT1	C-type lectin protein	KHN72314.1	C-type lectin-like	IPR001304	0.0717
A0A915APJ9	C-type lectin protein	KHN72313.1	C-type lectin-like	IPR001304	0.0667
A0A915AMS5	C-type lectin protein	VDM38028.1	C-type lectin-like	IPR001304	0.0581
A0A915AGW4	Maltase-glucoamylase	KHN72295.1	Glycoside hydrolase family 31	IPR000322	0.0558
A0A915BFK6	actin 2	KHN87379.1	Actin family	IPR004000	0.0497
A0A914ZHN3	Maltase-glucoamylase	KHN77140.1	Glycoside hydrolase family 31	IPR000322	0.0478
A0A914ZFS2	Sucrase-isomaltase	KHN73901.1	Glycoside hydrolase family 31	IPR000322	0.0438
A0A914ZGG9	unnamed protein product	VDM37827.1	Glycoside hydrolase family 31	IPR000322	0.0397
A0A915AHV5	C-type lectin protein	KHN84361.1	C-type lectin-like	IPR001304	0.0361
A0A915ALF1	C-type lectin protein	KHN84361.1	C-type lectin-like	IPR001304	0.0331
Adult male-derived EVs					
A0A915AGW4	Maltase-glucoamylase	KHN72295.1	Glycoside hydrolase family 31	IPR000322	0.1014
A0A914ZFS2	Sucrase-isomaltase	KHN73901.1	Glycoside hydrolase family 31	IPR000322	0.0737
A0A914ZHN3	Maltase-glucoamylase	KHN77140.1	Glycoside hydrolase family 31	IPR000322	0.0625
A0A914ZGG9	unnamed protein product	VDM37827.1	Glycoside hydrolase family 31	IPR000322	0.0606
A0A915BFK6	actin 2	KHN87379.1	Actin family	IPR004000	0.0536
A0A915C915	unnamed protein product	VDK19334.1	Domain of unknown function DUF4440	IPR027843	0.0505
A0A915APJ9	C-type lectin protein	KHN72313.1	C-type lectin-like	IPR001304	0.0422
A0A915BW41	hypothetical protein	KHN85026.1	von Willebrand factor, type A	IPR002035	0.0413
A0A915ALF1	C-type lectin protein	KHN72314.1	C-type lectin-like	IPR001304	0.0367
A0A915CAV2	unnamed protein product	VDK19334.1	Domain of unknown function DUF4440	IPR027843	0.0251

*NSAF* Normalized spectral abundance factor

**Fig. 4:**
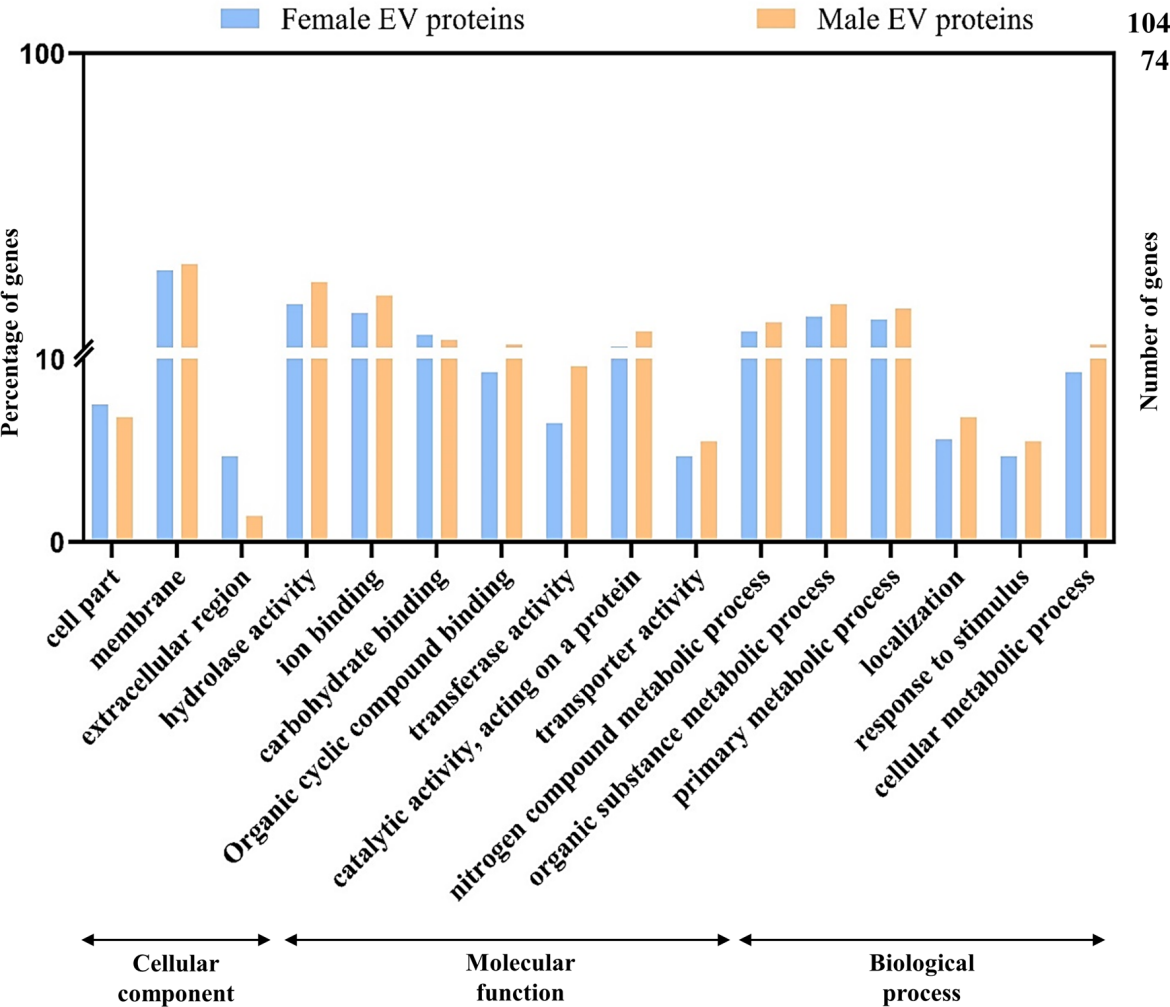
Gene Ontology (GO) classification of extracellular vesicle (EV) proteins between adult male and adult female *Parascaris* spp. The differentially expressed proteins are categorized into three hierarchical GO terms: biological process, cellular component, and molecular function. The y-axis represents the number and percentage of proteins in each respective GO term

Gene Ontology terms were assigned to the EV proteins identified from the male (62/74) and female (84/104) *Parascaris* spp. A summary of the comparison of male and female vesicle protein GO annotations categorized according to cellular component, molecular function, and biological process is shown in Fig. 4. A high degree of similarity in GO term allocation was observed between EVs derived from male and female worms. The most represented GO terms in the biological process category in the female and male EV proteins were “organic substance metabolic process” (GO:0071704), “primary metabolic process” (GO:0044238), and “nitrogen compound catabolic process” (GO:0006807). Similarly, the most represented GO terms within the molecular function category were “hydrolase activity” (GO:0016787), “ion binding” (GO:0043167), and “carbohydrate binding” (GO:0030246) and in the cellular component category, most of the proteins were assigned to “membrane” (GO:0016020), “cell part” (GO:0044464) and “extracellular region” (GO:0005576) (Additional file 3: Table S1).

KEGG pathway analysis using GhostKOALA resulted in the annotation of 45.2% (47/104) and 56.8% (42/74) EV proteins derived from female and male *Parascaris* spp., respectively. Several pathways related to metabolic, genetic, environmental, and cellular processes were shared between the male and female *Parascaris* spp. EV proteins. These pathways included starch and sucrose metabolism (ko00500), galactose metabolism (ko00052), glutathione metabolism (ko00480), as well as pathways involved in cellular structures and processes such as phagosome formation (ko04145), and lysosome function (ko04142) among others. Despite these observed similarities, there were differences in the signal transduction pathways between male and female *Parascaris* EV proteins. In females, unique proteins such as RHOA (Ras homolog gene family, member A) and selectin were associated with the Wnt (ko04310), TGF-Beta (ko04350), and TNF (ko04668) signaling pathways. In contrast, in males, proteins like KRAS (GTPase KRas) and 14–3-3 protein were specifically identified and linked to the Apelin (ko04371) and FoxO (ko04068) signaling pathways. Among the pathways that are involved in cell growth and death, proteins involved in cell cycle, meiosis, apoptosis, and senescence were identified in in males, whereas only the apoptosis pathway was represented in females, highlighting sex-specific variations. A detailed description of the KEGG orthologs of the male and female *Parascaris* EV proteins is listed in Additional file 3: Table S2.

## Discussion

In this study, we confirmed that adult *Parascaris* spp. secrete EV-like vesicles into the extracellular milieu. Proteomic profiling of EVs derived from adult males and females established the identity of the protein cargo of these vesicles and determined their putative functions. Particle size determination by NTA analysis and EM visualization of EVs with diameters ranging from 50 to 200 nm provided robust evidence that the purified vesicles were indeed extracellular vesicles. Most EV proteins showed *T. canis* protein homologues as their top similarity, primarily due to the absence of the *Parascaris* spp. protein data in the NCBI database. Silver staining of the EV lysate suggested that adult *Parascaris* EVs packed a heterogeneous mixture of protein and that potential differences existed in the protein cargo between the EVs of male and female *Parascaris* spp. Furthermore, proteomic data revealed a notable difference in the complexity of the extracellular vesicle (EV) proteomes of male and female *Parascaris* spp. This pattern of greater protein complexity in female EVs than in male EVs has also been observed in the secretomes and EVs of *Brugia* [15, 29]. Female parasites also tend to secrete a greater quantity of EV proteins, and one potential explanation for this sex-based disparity in EV cargo could be the origin and release location of the EVs. The site of EV generation and release may differ between males and females to address reproductive needs. These distinct locations could lead to variations in the types and quantities of proteins packaged within EVs. The specific factors underlying this difference in EV cargo between male and female worms warrant further investigation.

The abundant EV proteins identified from both male and female *Parascaris* spp. belong to the C-type lectin family and glycosidases. Several C-type lectin proteins have been identified in helminths and play diverse roles, such as binding to host ligands [30], exhibiting antibacterial properties [31], influencing reproduction [32], and interfering with mucin production by the host [33, 34]. These functions of the C-type lectin might facilitate the successful establishment of the parasite within the gut, promote survival, and evade the innate defenses of the host. Certain C-type lectins were specifically present in the EVs of female *Parasascaris* spp., while others were relatively abundant compared to male-derived EVs. The greater abundance of C-type lectins in female-derived EVs could be attributed to specific functions related to the female reproductive biology of *Parascaris*, such as regulating egg production or evasion of the host's immune defenses, allowing the parasites to establish and maintain infection. Further research is needed to fully understand the underlying mechanisms and evolutionary advantages of these sex-specific protein production patterns.

Maltase-glucoamylase and glyceraldehyde 3-phosphate dehydrogenase (GAPDH) were identified in *Parascarsis* EVs. Canonically, glycolytic enzymes support nutritional needs by facilitating the breakdown of complex carbohydrates. However, the release of glycosidases through EVs into the microenvironment of their predilection site could possibly suggest diverse roles in host-parasite interactions [35–37]. A GAPDH secreted by *Haemonchus contortus* was shown to aid evasion of the innate immune response by inhibiting complement activation pathways and thus preventing the formation of the membrane attack complex [36, 38]. Similarly, by degrading intestinal mucin, glycosidases can promote the establishment of infection, parasite development and prevent worm expulsion [35, 37].

Gut-dwelling adult nematodes live in a microbiota-rich environment and may require interactions with these communities. The molecular mechanism involved in this interaction is unclear, but proteins with potential antimicrobial activities, such as antibacterial factor (ASABF), C-type lectins, and galectins, have been identified in *A. suum* [39] and *liver flukes * [40, 41]. Proteomic analysis of *Parascaris* EV cargo also revealed homologs of these C-type lectins, galectins, and ASABF. C-type lectins are abundant in the protein cargo of *C. elegans* EVs, [42] and it has been shown that infection of *C. elegans* with the Gram-negative bacterium *Serratia marcescens,* significantly upregulated the expression of genes encoding lectins and lysozymes, emphasizing the potential involvement of these proteins in anti-bacterial immune defense [31]. Similarly, EV-associated lectins from parasitic nematodes could contribute to the modulation of bacterial communities and the local immune response in the host gut.

Members of the heat shock protein (HSP) family are molecular chaperones that aid in protein folding, maturation, and stability. Large HSPs, such as those in the HSP90 and HSP70 families, are expressed both constitutively and in response to stress. Thus, they play roles in both development and stress response and assist parasites in adapting to rapidly changing environments [43, 44]. HSP70, a known EV marker, was identified in *Parascaris*-derived EVs. HSP70 derived from helminths has shown promise as a vaccine candidate due to its immunogenic properties [45].

As in several species of nematodes, homologs of macrophage migration inhibitory factor (MIF) were detected in *Parascaris* spp. Immunolocalization studies have demonstrated the presence of MIFs on nematode surfaces and in the lining of the gut [46], suggesting that they could be part of the EV cargo. Nematode MIFs mimic host cytokines, and studies have shown that nematode MIFs can affect the migration of immune cells to create an anti-inflammatory environment that is favorable for parasite survival [47, 48].

Several proteins involved in EV biogenesis and structural proteins, such as tetraspanins, annexins, small GTPase-rab, profilin, and plastin, were identified. The presence of a CD36-like class B scavenger receptor that belongs to the tetraspanin family is associated with the acquisition of host lipids in parasites such as *Schistosoma mansoni* and *Opisthorchis viverrini* [49, 50], which are believed to serve nutritional, developmental, and possibly immune evasion purposes [51]. Considering the presence of this homologous protein in *Parascari*s EVs, it is plausible that it may be involved in improving lipid uptake, thus promoting parasite growth. Annexins are a group of calcium-dependent phospholipid-binding proteins that have been suggested to play roles in various critical biological processes. In the metacestode stage of *Taenia solium* infection*,* annexins help to evade the host immune response by inducing eosinophil apoptosis [52].

Generally, tetraspanins are considered molecular markers of EVs due to their presence on the surface membrane of EVs. Trematode EVs are distinguished by a significant amount of tetraspanins, but these proteins are less commonly identified from nematode-derived EVs. For instance, tetraspanins were absent in EVs of *A. suum* [9], *B. malayi* [15, 53] and *T. circumcincta* [12], whereas nematodes such as *Nippostrongylus brasiliensis* [14], *Heligmosomoides polygyrus* [54], and *Trichuris muris* [55] were reported to possess one or two tetraspanins in their EVs. Similarly, only one protein belonging to the tetraspanin family (A0A915BTK2_PARUN) was detected in *Parascaris* extracellular vesicles.

The Gene Ontology analysis of *Parascaris* EV revealed a significant representation of catalytic and hydrolase activities, which signifies the presence of enzymes involved in various metabolic processes. Parasites require these enzymes to break down complex molecules into simpler forms to aid in nutrient acquisition and energy production. These proteases serve multiple crucial functions, including metabolic food processing, inhibiting the antibody-dependent cell cytotoxicity (ADCC) response through IgG degradation [56] and modulating the host immune system by targeting immune cell receptors and mucins [57]. The identification of pathways related to carbohydrate metabolism pathways underscores that *Parascaris* spp. actively metabolize carbohydrates and highlights the importance of these processes in parasite survival. Carbohydrate metabolism is vital for energy production and the biosynthesis of essential molecules, indicating the parasite's dependence on carbohydrates for its biological activities [58].

## Conclusions

Overall, this study established the protein composition of extracellular vesicles (EVs) released by adult *Parascaris* spp. and discussed the differences in EVs derived from male and female *Parascaris.* The proteins carried by *Parascaris* EVs could play a significant role in the interaction between the parasite and its host. The ability of these EV proteins to potentially interfere with the host immune response indicates an advanced survival mechanism employed by the parasites, enabling them to persist within the host for extended periods without being expelled. This study not only enhances our understanding of parasite biology but also opens avenues for investigating parasite EVs and their constituents as potential tools for diagnosis and infection control.

## Supplementary Information

Additional File 1. Table S1. List of protein identities established through BLAST homology searches: Table S2. List of protein identities established through InterPro homology searches

Additional File 2. Table S1. Proteins of the extracellular vesicles of male *Parascaris* spp. arranged based on the abundance. Table S2. Proteins of the extracellular vesicles of female *Parascaris* spp. arranged based on the abundance.

Additional File 3. Table S1: Gene Ontology terms assigned to the EV proteins identified from the male and female *Parascaris* spp. Table S2: A detailed description of the KEGG orthologs of the male *Parascaris *EV proteins. Table S3: A detailed description of the KEGG orthologs of the female *Parascaris *EV proteins.

## Data Availability

The data generated has been deposited into the public respository, MassIVE, MSV000095723.

## Abbreviations

ADCC: Antibody-dependent cell-mediated cytotoxicity
ASABF: Ascaris suum anti-bacterial factor
BCA: Bicinchoninic acid
BLAST: Basic local alignment search tool
CD-36: Cluster of differentiation 36
DMEM: Dulbecco's modified eagle's medium
DTT: Dithiothreitol
EVs: Extracellular vesicles
GAPDH: Glyceraldehyde-3-phosphate dehydrogenase
GO: Gene ontology
GTP: Guanosine triphosphate
HSP70: Heat shock protein 70
KEGG: Kyoto encyclopedia of genes and genomes
LC–Ms/MS: Liquid chromatography tandem mass spectrometry
NSAF: Normalized spectral abundance factor
NTA: Nanoparticle tracking analysis
PBS: Phosphate buffered saline
SDS-PAGE: Sodium dodecyl sulfate–polyacrylamide gel electrophoresis
TEM: Transmission electron microscopy
